# Validation of real-time RT-PCR for detection of SARS-CoV-2 in the early stages of the COVID-19 outbreak in the Republic of Korea

**DOI:** 10.1038/s41598-021-94196-3

**Published:** 2021-07-20

**Authors:** Yoon-Seok Chung, Nam-Joo Lee, Sang Hee Woo, Jeong-Min Kim, Heui Man Kim, Hye Jun Jo, Ye Eun Park, Myung-Guk Han

**Affiliations:** 1 Division of Infectious Disease Diagnosis Control, Honam Regional Centers for Disease Control and Prevention, Korea Diseases Control and Prevention Agency, Gwangju-si, 61947 Republic of Korea; 2 Division of Emerging Infectious Diseases, Bureau of Infectious Disease Diagnosis Control, Korea Diseases Control and Prevention Agency, Cheongju-si, 28159 Republic of Korea; 3 Division of Laboratory Diagnosis Management, Bureau of Infectious Disease Diagnosis Control, Korea Diseases Control and Prevention Agency, Cheongju-si, 28159 Republic of Korea; 4 Division of Viral Diseases, Bureau of Infectious Disease Diagnosis Control, Korea Diseases Control and Prevention Agency, 187 Osongsaengmyeong2-ro, Osong-eup, Heungdeok-gu, Cheongju-si, 28159 Republic of Korea

**Keywords:** Clinical microbiology, Virology

## Abstract

A real-time reverse transcription polymerase chain reaction (RT-qPCR) assay that does not require Emergency Use Authorization (EUA) reagents was tested and validated for the detection of severe acute respiratory syndrome coronavirus 2 (SARS-CoV-2) during the early stages of the outbreak of coronavirus disease 2019 (COVID-19) in the Republic of Korea. Early diagnosis of COVID-19 enables timely treatment and the implementation of public health measures. We validated the sensitivity, specificity, precision, linearity, accuracy, and robustness of the RT-qPCR assay for SARS-CoV-2 detection and compared its performance with that of several EUA-approved kits. Our RT-qPCR assay was highly specific for SARS-CoV-2 as demonstrated by not amplifying 13 other viruses that cause respiratory diseases. The assay showed high linearity using a viral isolate from a patient with known COVID-19 as well as plasmids containing target SARS-CoV-2 genes as templates. The assay showed good repeatability and reproducibility with a coefficient of variation of 3%, and a SARS-CoV-2 limit of detection of 1 PFU/mL. The RT-qPCR-based assay is highly effective and can facilitate the early diagnosis of COVID-19 without the use of EUA-approved kits or reagents in the Republic of Korea.

## Introduction

Coronavirus disease 2019, officially named COVID-19 by the World Health Organization (WHO) is a severe acute respiratory syndrome (SARS) caused by the novel severe acute respiratory syndrome coronavirus 2 (SARS-CoV-2). COVID-19 was first reported as an idiopathic pneumonia in Hubei Province, Wuhan, China, in December 2019^1^. SARS-CoV-2 is more contagious than SARS-CoV, which was first reported in China in 2002, and Middle East respiratory syndrome coronavirus (MERS-CoV), which first emerged in the Middle East in 2012^2^. SARS-CoV-2 has since spread to several countries outside of China, affecting populations worldwide. On March 11, 2020, the WHO declared COVID-19 to be a pandemic, and by April 24, 2020, the number of confirmed cases of COVID-19 reached 2,653,573 and the number of deaths reached 189,658, across 181 countries. In South Korea, the first confirmed case was reported on January 19, 2020, followed by a surge in confirmed cases on February 19; by April 24, there were 10,718 confirmed cases and 240 deaths reported across the Republic of Korea^3–6^.

SARS-CoV-2 belongs to the subfamily *Orthocoronavirinae*, which is a member of the family *Coronaviridae*^7^. This beta coronavirus has a 30-kb genome, sharing 96% sequence identity with the bat coronavirus RaTG13, 88% with bat coronaviruses ZC45 and ZXC21, 80% with SARS-CoV, and 50% with MERS-CoV^8^. Coronaviruses are enveloped, positive-sense, single-stranded RNA viruses, with at least six open reading frames. The coronavirus genome encodes the structural glycoproteins spike (S), membrane (M), envelope (E), and nucleocapsid (N)^8^.

Real-time reverse transcription polymerase chain reaction (RT-qPCR) is widely used to detect gene expression levels, while also facilitating the rapid diagnosis of acute respiratory viral infections^9^. COVID-19 can be diagnosed in the laboratory by detecting SARS-CoV-2 genes in clinical samples collected from suspected patients, followed by viral isolation and culture^10^. RT-qPCR is commonly used worldwide to diagnose COVID-19 in the laboratory setting^9,11,12^.

An RT-qPCR-based assay for detecting SARS-CoV-2 was first developed at the Charité Institute of Virology in Germany, and introduced by the WHO on January 13, 2020^9^. Additional protocols were subsequently reported by the Chinese Center for Disease Control and Prevention, the University of Hong Kong, and the Centers for Disease Control and Prevention of the United States^13^. The assay targets the SARS-CoV-2 RNA-dependent RNA polymerase (RdRp) gene, as well as the E and N genes^14^. Published protocols differ based on the target genes, primer and probe sequences, mixture composition, amplification conditions, sensitivity, and the requirement for Emergency Use Authorization (EUA)-approved reagents. This was especially true during the early outbreak of COVID-19 in Korea when most protocols required the use of EUA-approved specific reagents for RT-qPCR, some of which were not available in Korea. A standardized and validated assay, exhibiting highly accurate laboratory performance without the need for EUA-approved reagents, for the detection of SARS-CoV-2 is essential. In this study, we assessed the analytical sensitivity, specificity, precision, linearity, accuracy, and robustness of the RT-qPCR-based assay we developed, which did not require EUA-approved reagents, for the detection of SARS-CoV-2. We also compared the accuracy of our assay results with those obtained using five different COVID-19 EUA-approved kits and respiratory samples from patients with and without suspected COVID-19.

## Methods

### Ethics approval and consent to participate

This study was approved by the Korea Centers for Diseases Control and Prevention Ethics Committee—KCDC Authority (approval number #2020-03-01-P-A). The requirement for informed consent was waived by Korea Centers for Diseases Control and Prevention Research Ethics Committee as this study was part of a public health surveillance and outbreak investigation in Republic of Korea. This study was performed in accordance with the relevant laws and regulations that govern research in the Korea Centers for Diseases Control and Prevention.

### Cells and viruses

Vero E6 cells were inoculated with the SARS-CoV-2/Korea/KCDC03/2020 virus, isolated by the Korean Center for Disease Control, and cultured for 4 d. The culture medium was then centrifuged, aliquoted, and stored at -70 °C. Virus titers were measured using a plaque assay. Viral culture was performed in a biosafety level (BSL)-3 laboratory.

### Extraction of viral RNA

RNA was extracted from the culture medium (140 µL) containing the SARS-CoV-2/Korea/KCDC03/2020 virus using a QIAamp Viral RNA Mini kit (QIAGEN, Hilden, Germany) according to the manufacturer’s instructions. Viral lysis was performed in a BSL-3 laboratory, whereas procedures involving RNA were performed in a BSL-2 laboratory.

### RT-qPCR for SARS-CoV-2 detection

Sequences of primers and probes, provided by the WHO, used in RT-qPCR^2^ are shown in Table 1. AgPath-ID one-step RT-PCR reagents (Applied Biosystems, Foster City, CA, USA) were used in accordance with the manufacturer’s instructions. The viral RNA sample (5 µL) was mixed with the RT-PCR reagents and the corresponding RdRp or E gene primers (1 µL, 10 pmol) and probe (0.5 µL , 10 pmol). PCR was performed at 50 °C for 30 min, 95 °C for 10 min, 95 °C for 15 s, and 60 °C for one min, for 40 cycles; carboxyrhodamine (ROX) was used as a passive reference dye. The Applied Biosystems 7500 Fast Real-Time PCR System was used for RT-qPCR, and the cycle threshold (Ct) value of the SARS-CoV-2 target gene was ascertained (Table 1).

**Table 1:** Primers and probes used to detect SARS-CoV-2.

	Primer/Probe	Sequence (5'–3')
RdRp gene	RdRp_SARSr-F2	GTGARATGGTCATGTGTGGCGG
	RdRp_SARSr-R1	CARATGTTAAASACACTATTAGCATA
	RdRp_SARSr-P2	FAM-CAGGTGGAACCTCATCAGGAGATGC-BHQ
E gene	E_Sarbeco_F1	ACAGGTACGTTAATAGTTAATAGCGT
	E_Sarbeco_R2	ATATTGCAGCAGTACGCACACA
	E_Sarbeco_P1	FAM-ACACTAGCCATCCTTACTGCGCTTCG-BHQ

R is G/A; FAM, 6-carboxyfluorescein; BHQ, black hole quencher.

### Determination of specificity and sensitivity

To assess the specificity of the RT-qPCR assay, 23 virus strains—human coronavirus 229E, NL63, OC43, HKU1, MERS-CoV, influenza virus A/H1N1pdm09, A/H3N2, B, adenovirus type 5, rhinovirus, parainfluenza virus 1/2/3, respiratory syncytial virus A/B, metapneumovirus, bocavirus, measles virus, mumps virus, rubella virus, enterovirus, varicella-zoster virus, and hantavirus (Table 2)—and five samples showing negative results for a known respiratory virus were used as template.

**Table 2:** SARS-CoV-2 (COVID-19) nucleic acid detection kits with emergency use approval (EUA) in the Republic of Korea.

Product name	Approval date	Target gene	Manufacturer
PowerCheckTM2019-nCoV	Feb. 4. 2020	RdRp, E	Kogenbiotech
AllplexTM2019-nCoVAssay	Feb.12. 2020	RdRp, E, N	Seegene
DiaPlexQTMNovel Coronavirus (2019-nCoV) Detection Kit	Feb.27. 2020	ORF1a, N	Solgent
STANDARD M nCoV Real-Time Detection Kit	Feb.27. 2020	RdRp, E	SD Biosenser
Real-Q 2019-nCoV Detection kit	Mar.13. 2020	RdRp, E	Bioseum

Sensitivity of the RT-qPCR assay was measured by RT-qPCR using plasmids containing cloned target SARS-CoV-2 genes (RdRp and E), which were serially diluted tenfold from different initial concentrations. To examine the responsivity of the assay, RT-qPCR was also performed using tenfold serially diluted RNA extracted from a lower respiratory tract sample of the first patient who tested positive for COVID-19 in Republic of Korea.

### Plaque assay for virus titration

Vero E6 cells were seeded into 12-well plates at 2.0 × 10^5^ cells per well. After 24 h, the cells were infected with 50 μL of tenfold serial dilutions of the isolated viruses, and incubated for 1 h to facilitate viral adsorption. The cells were then covered with a basal Minimal Essential Media (MEM)-α agar overlay containing 0.02% (w/v) diethylaminoethyl-dextran, 0.1% (w/v) glucose, 0.7% (w/v) SeaKem LE Agarose (LONZA, Basel, Switzerland), 30 mM MgSO_4_, and 4 μg/mL trypsin (Gibco, Grand Island, NY, USA). The cells were incubated at 37 °C for 3 d to facilitate infection. Two days after viral infection, a 0.03% (w/v) crystal violet overlay was added to each well to stain viable cells.

### Intra- and inter-assay reproducibility and efficiency

To determine the limit of detection (LOD), the titers of the isolated viruses were measured in plaque-forming units (PFU). The virus culture medium was diluted from 3.45 × 10^6^ PFU/mL to 1 × 10^5^ PFU/mL. Ten-fold dilutions were prepared until a concentration of 1 × 10^−2^ PFU/mL was obtained. RNA was extracted from each diluent and used for RT-qPCR targeting the SARS-CoV-2 RdRp and E genes. RT-qPCR was performed in triplicate to assess the assay reproducibility, and the assay was repeated 3 d later using RNA extracted from the diluted virus culture media to assess repeatability.

### Determination of accuracy of EUA kits

To investigate the accuracy of the COVID-19 EUA-approved kits in the Republic of Korea (Table 2), 55 positive samples (selected five-step positive samples based on the distribution of RdRp gene Ct values) and 50 negative samples were analyzed. The results (sensitivity and specificity) were compared with those from our RT-qPCR assay of the same samples.

### Statistical analysis

Inter-assay and intra-assay variations in the Ct value were determined for the triplicate RT-qPCR reactions and for the repeat assay 3 d later. The reliability of each experiment was determined from the F and *P* values.

## Results

### Specificity and sensitivity

Viral RNA specific to human coronaviruses 229E, NL63, OC43, and HKU1; SARS-CoV; MERS-CoV; influenza virus; adenovirus; rhinovirus; parainfluenza virus; respiratory syncytial virus; metapneumovirus; and bocavirus was not detected in the RT-qPCR specificity assay using primers targeting SARS-CoV-2 RdRp and E genes (Table 3). The Ct value could not be determined for measles virus, mumps virus, rubella virus, enterovirus D68, nor the known-negative nasopharyngeal swab specimens. These results indicate that the assay was highly specific for both of the SARS-CoV-2 target genes RdRp and E.

**Table 3:** Specificity evaluation of the SARS-CoV-2 RT-qPCR assay using known respiratory viruses and respiratory specimens and primers to the SARS-CoV-2 RdRp and E genes.

Viruses and specimens	Subtype strain	Real-time RT-PCR (Ct value)	
		RdRp	E
HCoV 229E		UD	UD
HCoV NL63		UD	UD
HCoV OC43		UD	UD
HCoV HKU1		UD	UD
MERS-CoV	KCDC	UD	UD
Influenza virus	A(H1N1)	UD	UD
Influenza virus	A(H3N2)	UD	UD
Influenza virus	B	UD	UD
Adenovirus	Type 5	UD	UD
Rhinovirus		UD	UD
Parainfluenza virus	1	UD	UD
Parainfluenza virus	2	UD	UD
Parainfluenza virus	3	UD	UD
Respiratory syncytial virus	A	UD	UD
Respiratory syncytial virus	B	UD	UD
Human metapneumovirus		UD	UD
Human bocavirus		UD	UD
Measles virus	A	UD	UD
Mumps virus	Jerylin	UD	UD
Rubella virus	Moraten	UD	UD
Enterovirus	D68	UD	UD
Varicella-zoster virus		UD	UD
Hantanvirus		UD	UD
Negative specimen 1		UD	UD
Negative specimen 2		UD	UD
Negative specimen 3		UD	UD
Negative specimen 4		UD	UD
Negative specimen 5		UD	UD

UD, Undetected; negative specimen, specimens known to be negative for SARS-CoV-2.

**Figure 1:**
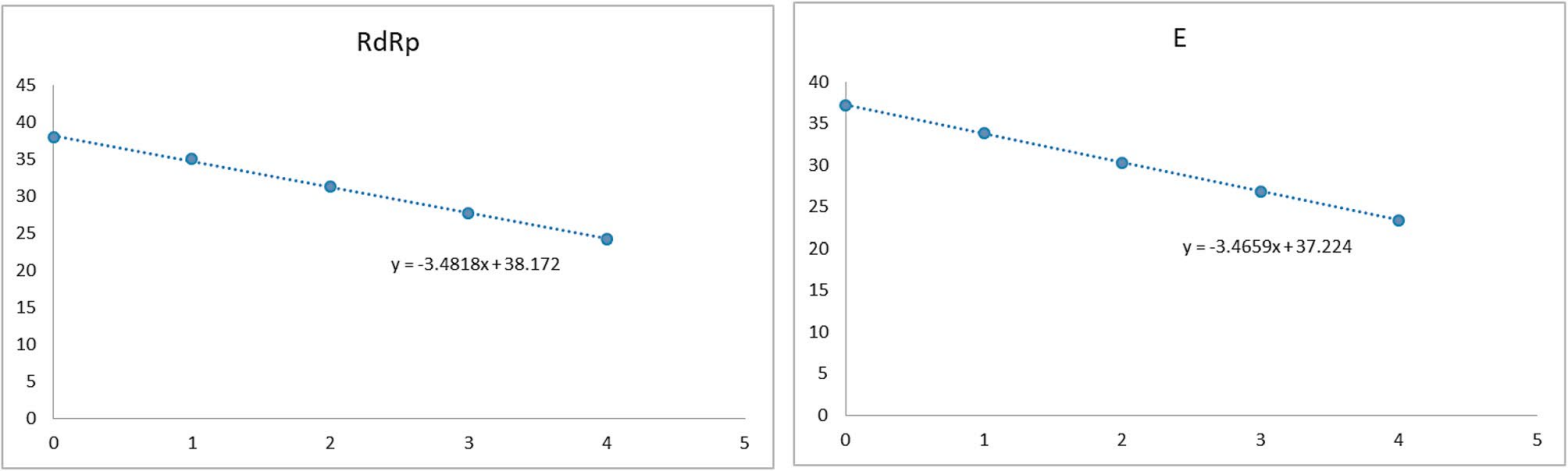
Analysis of linearity of RT-qPCR results targeting SARS-CoV-2 RdRp and E genes in plasmid DNA containing cloned target sequences.

Analytical sensitivity of the RT-qPCR assay was assessed by determining the LOD for each gene using plasmid DNA containing the SARS-CoV-2 RdRp or E gene. The assay had a mean LOD of 8 × 10^0^ copies/mL in triplicate runs (Table 4). Averaged Ct values of 37.96 and 37.19 and coefficient of variation (CV) values of 0.65 and 0.34 were obtained for the RdRp and E genes, respectively, indicating good analytical performance (Table 4; Fig. 1).

**Table 4:** Sensitivity and repeatability of RT-qPCR amplification of SARS-CoV-2 plasmid cloned RdRp and E genes.

Plasmid gene (copies/mL)	1st		2nd		3rd		Average		CV	
	RdRp	E	RdRp	E	RdRp	E	RdRp	E	RdRp	E
8 × 10^4^	24.18	23.51	24.25	23.38	24.15	23.28	24.19	23.39	0.05	0.12
8 × 10^3^	27.68	26.73	27.78	26.79	27.62	26.88	27.69	26.80	0.08	0.07
8 × 10^2^	31.28	30.10	31.24	30.29	31.14	30.31	31.22	30.23	0.07	0.11
8 × 10^1^	35.41	33.74	35.00	33.90	34.51	33.92	34.98	33.85	0.45	0.10
8 × 10^0^	37.21	37.56	38.26	36.88	38.41	37.13	37.96	37.19	0.65	0.34
8 × 10^−1^	UD	UD	UD	UD	UD	UD	UD	UD	UD	UD
8 × 10^−2^	UD	UD	UD	UD	UD	UD	UD	UD	UD	UD
Negative control	UD	UD	UD	UD	UD	UD	UD	UD	UD	UD

1st, 2nd, and 3rd refer to three independent analyses of each plasmid sample; CV, coefficient of variation; UD, Undetected.

### Evaluation of assay efficacy using a sample from the first patient with confirmed COVID-19 in Republic of Korea

**Figure 2:**
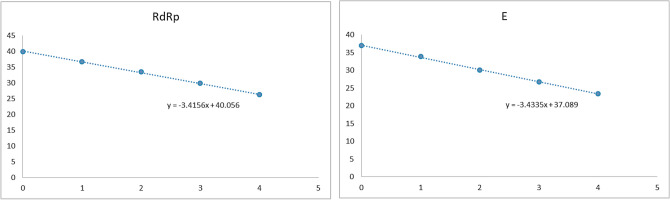
Analysis of linearity of RT-qPCR results targeting SARS-CoV-2 RdRp and E genes in RNA isolated from a nasopharyngeal swab used in testing the first patient confirmed to have COVID-19 in South Korea.

RNA was extracted from a lower respiratory tract mucus sample from the first patient confirmed to have COVID-19 in South Korea. RT-qPCR was performed after tenfold serial dilution of the RNA sample to evaluate the responsivity and efficacy of the assay^4^. The Ct value of the SARS-CoV-2 RdRp gene was 36.62 at a 10^−4^-fold dilution, and that of the E gene was 36.97 at 10^−5^-fold dilution (Fig. 2). RT-qPCR accurately detected the target genes in the patient sample.

### Linearity of RT-qPCR for detecting SARS-CoV-2

**Figure 3:**
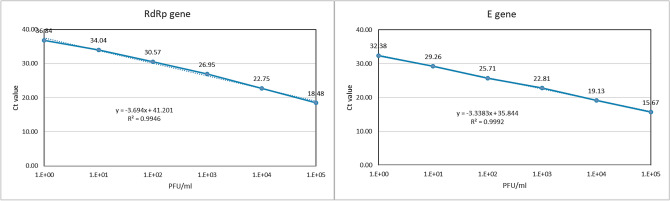
Analysis of linearity of RT-qPCR results targeting SARS-CoV-2 RdRp and E genes in RNA form virus isolates.

Assay performance was assessed using tenfold serial dilutions of virus with known PFUs as standards for the consensus sequence and each isolate of SARS-CoV-2 in South Korea. Four independent runs were performed. Linear regression analysis revealed correlation coefficients of R^2^ = 0.998 among viruses with known PFU values (Fig. 3).

### Limit of detection and limit of quantification (correlation between RT-qPCR and virus titration)

To determine the LOD for SARS-CoV-2, tenfold serially diluted plasmid DNA containing either the RdRp or E target gene was used as template in three independent RT-qPCR runs. The analytical detection limit was 8 × 10^0^ copies/mL for all RT-qPCR assays (Table 4, Fig. 1). RNA was extracted from viral culture medium of a known viral titer, following serial dilution from 10^5^ to 10^−2^ PFU/mL. RT-qPCR was repeated four times using the RNA template to determine the limit of quantification at each concentration. The LOD was 1 PFU/mL (Table 5, Fig. 3).

**Table 5:** Accuracy and precision of RT-qPCR amplification of SARS-CoV 2 RdRp and E genes from a lower respiratory tract mucus sample from the first patient confirmed to have COVID-19 in South Korea.

SARS-CoV-2 PFU	Inter CV%	F-value	*P*-value	Intra CV%
**A. RdRP gene**				
1 × 10^5^	2.47	0.98	0.36	0.64
1 × 10^4^	2.49	0.11	0.75	1.02
1 × 10^3^	2.56	0.61	0.46	0.75
1 × 10^2^	2.71	0.21	0.66	1.21
1 × 10^1^	2.68	0.00	0.99	0.26
1 × 10^0^	1.88	0.35	0.57	0.74
**B. E gene**				
1 × 10^5^	3.47	0.01	0.93	0.43
1 × 10^4^	2.10	0.01	0.91	0.39
1 × 10^3^	2.42	0.00	0.95	0.16
1 × 10^2^	1.55	0.00	0.98	0.20
1 × 10^1^	1.39	0.00	1.00	0.74
1 × 10^0^	0.71	1.36	0.29	1.02

*P* value > 0.05 indicates no difference between days. *F* value < 5.99 indicates no difference between days. F(1,6; 0.05) = 5.99; CV(%): Coefficient of variation.

### Accuracy and precision

To assess the accuracy and precision of detecting SARS-CoV-2 target genes using the RT-qPCR assay, four tenfold serial dilutions of a virus culture medium with a known virus titer were analyzed and the experiment was repeated 3 d later. The inter-assay CV was 1.88–2.71 and 0.71–3.47, whereas the intra-assay CV was 0.26–1.21 and 0.16–1.02 for the RdRp and E genes, respectively. The *P* value was greater than 0.05, and the F value was smaller than 5.99 for all of the experiments, indicating accurate and precise detection of the genes (Table 5, Fig. 3).

### Comparison of RT-qPCR assay and EUA kits

All five of the nucleic acid detection kits evaluated in this study could detect SARS-CoV-2 in respiratory samples (known to be positive or negative for SARS-CoV-2) at a sensitivity of at least 98.2% and a specificity of 100% (95% confidence interval: 90.4–99.7%), when compared with the results of the RT-qPCR assay of this study. Inconsistent results obtained with one EUA kit were confirmed by further examination to be reflective of an inconclusive case rather than a false case (Table 6).

**Table 6:** Comparison of sensitivity and specificity of SARS-CoV 2 detection in respiratory samples using Emergency Use Authorization (EUA) PCR kits and the assay developed in this study.

This study		PowerCheck 2019-nCoV			Allplex 2019-nCoVAssay			DiaPlexQ Novel Coronavirus			STANDARD M nCoV Real-Time Detection kit			Real-Q 2019-nCoV Detection kit		
		Pos	Neg	Inc	Pos	Neg	Inc	Pos	Neg	Inc	Pos	Neg	Inc	Pos	Neg	Inc
Pos	54	53		1*	54			54			54			54		
Neg	50		50			50			50			50			50	
Inc	1			1			1			1			1			1
Sensitivity^¶^ (%)		98.2			98.2			98.2			98.2			98.2		
Specificity^#^ (%)		100			100			100			100			100		

*Inconsistent (Inc) results in one kit were confirmed by further examination to be an inconclusive case and not a false case.

^¶^95% confidence interval: 90.4 ~ 99.7%.

^#^95% confidence interval: 92.9 ~ 100%.

## Discussion

Molecular methods are more rapid, accurate, and sensitive for virus detection than culture methods. In this study, we established a consensus method using molecular tools for detecting SARS-CoV-2 that did not require the use of EUA-approved reagents or kits. Early diagnosis of SARS-CoV-2-infected patients is essential for controlling the dynamics of the COVID-19 pandemic.

Since its initial emergence in Wuhan, China in late 2019, COVID-19 has rapidly spread worldwide^2^. COVID-19 is caused by SARS-CoV-2, with its clinical symptoms including dyspnea, cough, and mild respiratory symptoms that progress to pneumonia. It is difficult to distinguish SARS-CoV-2 from other common respiratory viruses such as influenza viruses, because of their highly similar symptoms^8^. A genetic assay with high specificity is necessary to detect SARS-CoV-2.

During early viral spread, the WHO published a protocol for detecting SARS-CoV-2. The assay was developed by the Charité Research Organization in Germany on January 17, 2020^9,15^. This was the first genetic assay to be developed and released after the first report of the SARS-CoV-2 genome on January 11, 2020. We used this assay to rapidly detect SARS-CoV-2. The protocol involves a common reporter dye, 6-carboxyfluorescein, together with BlackBerry Quencher; however, as this quencher is not used in Korea, it was replaced with Black Hole Quencher during probe synthesis. We used this modified assay to detect the target genes RdRp and E.

To assess the specificity of the assay, which was performed using specifically designed primer probes, RT-qPCR was conducted on 23 respiratory viruses, including influenza viruses, and five respiratory tract samples that had previously tested negative for SARS-CoV-2. No respiratory viruses other than SARS-CoV-2 were detected. The RT-qPCR assay showed excellent specificity and sensitivity, including a low LOD.

Studies have shown that it is possible to accurately detect COVID-19 genes using detection reagents that have not received EUA. The Provincial Institute of Health and Environmental Research carried out regional training programs for COVID-19 diagnosis, which were particularly useful in emergency diagnostic situations in the early stages of the pandemic. COVID-19 diagnostic EUA reagents were approved for private sector use after comparative analysis.

## Conclusions

In conclusion, we evaluated an RT-qPCR assay in the Early Stages of the COVID-19 Outbreak without Emergency Use Authorization Reagents in the Republic of Korea. The assay exhibited high specificity and sensitivity for SARS-CoV-2 and good analytical performance using cloned SARS-CoV-2 genes and/or virus isolated from the first patient who tested positive for COVID-19 in Republic of Korea. The RT-qPCR of this study was used as a criterion for evaluating EUA kits in Republic of Korea.

## Data Availability

No datasets were generated or analyzed during the current study.
